# Comment on three papers about Hardy–Weinberg equilibrium tests in autopolyploids

**DOI:** 10.3389/fgene.2022.1027209

**Published:** 2022-10-04

**Authors:** David Gerard

**Affiliations:** Department of Mathematics and Statistics, American University, Washington, DC, United States

**Keywords:** double reduction, equilibrium, Hardy–Weinberg, hypothesis tests, polyploids, random mating

## 1 Introduction

One goal in the population genetics of autopolyploids, which are organisms with more than two sets of homologous chromosomes, is to model their genotype frequencies. This modeling presents a greater challenge in autopolyploids than in diploids because certain meiotic configurations in autopolyploids can result in a phenomenon known as double reduction is the co-migration of sister chromatid segments to the same gamete (Stift et al., 2010). Double reduction affects both the segregation frequencies of genotypes from individuals to their offspring (Mather, 1936; Fisher and Mather, 1943; Bever and Felber, 1992; Huang et al., 2019) and the equilibrium genotype frequencies of a panmictic population (Geiringer, 1949; Bennett, 1968; Bever and Felber, 1992; Huang et al., 2019). Testing if a population is in equilibrium, or merely exhibits random mating, is important for the same reasons as tests for Hardy–Weinberg equilibrium in diploids, namely, for 1) determining the mating system, 2) detecting segregation distortion, 3) detecting inbreeding, and 4) detecting genotyping errors (Gerard, 2022b).

Three similar articles which attempt to test for equilibrium and random mating were recently released: one for tetraploids (Sun et al., 2021), one for hexaploids (Wang et al., 2022), and one for octoploids (Wang et al., 2021). These three articles have numerous implementation mistakes, confuse random mating and equilibrium, confuse autopolyploids and allopolyploids (organisms with homoeologous subgenomes), and provide suboptimal testing approaches. The objectives of this study are to correct the authors’ mistakes (Section 2), provide examples of how random mating and equilibrium differ in autopolyploids (Section 3), provide examples of how allo- and autopolyploids differ (Section 4), and promote the better methods of Gerard (2022b) and Gerard (2022a) (Section 5).

This study requires a little notation before the issues are discussed here. Let ***q*** = (*q*_0_, *q*_1_, … , *q*_*K*_) be the genotype frequencies at a single biallelic locus for an autopolyploid population with ploidy *K*; that is, *q*_*k*_ is the proportion of individuals in the population with *k* copies of the minor allele. Let ***x*** = (*x*_0_, *x*_1_, … , *x*_*K*_) be the genotype counts in a random sample of $n=\sum_{k=0}^{K}x_{k}$ individuals. Then ***x*** is multinomially distributed with size *n* and probability vector ***q***. Under random mating, the genotype frequencies are (Gerard, 2022b)

$$q_{k}=\overset{\min\left(k,K/2\right)}{\underset{i=\max\left(0,k-K/2\right)}{\sum }}p_{i}p_{k-i},$$ (1)

where ***p*** = (*p*_0_, *p*_1_, … , *p*_*K*/2_) are the gamete frequencies of the population; that is, *p*_*k*_ is the proportion of gametes in the population that have *k* copies of the minor allele. Suppose that a population is randomly mating, then there exists a function *f*(***q***, *α*) = (*f*_0_ (***q***, *α*), …, *f*_*K*_ (***q***, *α*)) that updates the genotype frequencies from the current generation ***q*** to the next *f*(***q***, *α*). Here, *α* is called the double reduction rate, which is a property of meiosis in autopolyploids (Stift et al., 2010). If the population is at equilibrium, then the genotype frequencies follow

$$q=f\left(q,\alpha \right).$$ (2)

For each ploidy, there is a ***q*** that satisfies Eq. 2, which is called the “equilibrium genotype frequencies” (Huang et al., 2019). These frequencies are a function of the double reduction rate *α* and the allele frequency $r=\frac{1}{K}\sum_{k=0}^{K}kq_{k}$ , and have been calculated for ploidies less than or equal to ten (Huang et al., 2019). If *α* = 0, then these equilibrium genotype frequencies reduce to binomial proportions (Haldane, 1930),

$$q_{k}=\left({}_{k}^{K}\right)r^{k}{\left(1-r\right)}^{K-k}.$$ (3)

This study concerns tests for Eqs 1–3.

## 2 Implementation and coding errors

There are many logical and coding issues in the studies by Sun et al. (2021), Wang et al. (2021), and Wang et al. (2022). In this section, the ones that were found are listed. However, the code from the study by Sun et al. (2021) is not available, and the code from the study by Wang et al. (2021) and Wang et al. (2022) is verbose and sparsely documented, so there might be more implementation errors that were missed. In particular, the following were found: 1) an incorrect model for meiosis for autooctoploids that results in incorrect equilibrium genotype frequencies, 2) two instances of incorrect *χ* ^2^ test statistic calculations, 3) five instances of incorrect degrees of freedom calculations, and 4) two instances of using unknown parameters in an estimation scheme.

The model for meiosis in the study by Wang et al. (2021) is incorrect. This leads to incorrect equilibrium genotype frequencies in their “recursive” test for equilibrium, and thus an incorrect test for equilibrium. It can be determined that their model is incorrect by looking at what it implies when *α* = 0. In this case, the distribution of gamete dosages is known to follow a hypergeometric distribution (Table 1 from Haldane, 1930; Huang et al., 2019). If *X* is the parental genotype and *Y* is the gamete genotype, the reader can see this result by thinking of the probability of obtaining *Y* minor alleles out of *K*/2 chosen alleles from an individual with *K* total alleles and *X* total minor alleles. Therefore, the correct segregation frequencies are obtained *via*

$$\mathrm{Pr}\left(Y=y|X=x\right)=\frac{\left({}_{y}^{x}\right)\left({}_{K/2-y}^{K-x}\right)}{\left({}_{K/2}^{K}\right)}.$$ (4)

Table 1 shows that the model for meiosis from Table 1 of the study by Wang et al. (2021) does not equal the probabilities from Eq. 4 when *α* = 0, indicating that their model for meiosis is incorrect. It can be empirically observed that their equilibrium frequencies also do not equal binomial proportions when *α* = 0 (Supplementary Appendix S2), which they should (Haldane, 1930).

**TABLE 1:** Segregation frequencies for an autooctoploid when there is no double reduction, either according to Table 1 from the study by Wang et al. (2021) or according to the correct calculation using the hypergeometric distribution (Eq. 4). The two approaches are different, so the general model for meiosis in the study by Wang et al. (2021) is incorrect.

Parent genotype	Method	Gamete genotype				
		4	3	2	1	0
8	Wang et al. (2021)	1	0	0	0	0
8	Correct	1	0	0	0	0
7	Wang et al. (2021)	9/16	3/8	1/16	0	0
7	Correct	1/2	1/2	0	0	0
6	Wang et al. (2021)	225/784	45/98	87/392	3/98	1/784
6	Correct	3/14	8/14	3/14	0	0
5	Wang et al. (2021)	25/196	75/196	285/784	45/392	9/784
5	Correct	1/14	6/14	6/14	1/14	0
4	Wang et al. (2021)	9/196	12/49	41/98	12/49	9/196
4	Correct	1/70	16/70	36/70	16/70	1/70
3	Wang et al. (2021)	9/784	45/392	285/784	75/196	25/196
3	Correct	0	1/14	6/14	6/14	1/14
2	Wang et al. (2021)	1/784	3/98	87/392	45/98	225/784
2	Correct	0	0	3/14	8/14	3/14
1	Wang et al. (2021)	0	0	1/16	3/8	9/16
1	Correct	0	0	0	1/2	1/2
0	Wang et al. (2021)	0	0	0	0	1
0	Correct	0	0	0	0	1

The *χ* ^2^ statistics testing hypotheses Eq. 1 and Eq. 2 are implemented incorrectly in the study by Wang et al. (2022). The *χ* ^2^ statistic in Eq. 1 of the study by Wang et al. (2022) is correct in the study, but in their code, they left out the *N* term. This affects both their equilibrium testing results and their random mating results. This is known because this study reproduced their 6.602 and 6.649 values from page five of their article (Supplementary Appendix S3). Thus, their tests are improperly implemented.

The random mating test is implemented incorrectly in the study by Wang et al. (2022), even after the *N* term is included; that is, the authors calculate it differently than what they state in their article. Particularly, in their code, they first estimate the gamete frequencies *via* maximum likelihood, and then put the resulting genotype frequencies through the recursive formula to come up with equilibrium values. However, using this recursive formula just results in the same genotype frequencies as the equilibrium “recursive” test. So, the 6.602 value and the 6.649 value aforementioned are different merely because the authors ran the recursive relationship for a different number of iterations.

A total of five instances of incorrect degrees of freedom calculations were counted for the chi-squared tests in the studies by Sun et al. (2021), Wang et al. (2022), and Wang et al. (2021). These calculations are described in Supplementary Appendix S6. Thus, most of the test statistics from these three articles are compared to the incorrect null distribution, resulting in incorrect *p*-values.

The estimates of *α* are implemented incorrectly in the study by Wang et al. (2022). The authors do not modularize their code into functions, and this led to some logical errors. They have a variable in their simulations called alpha, that is, the true double reduction rate. Their code returns alpha1, that is, the estimated double reduction rate. However, their EM algorithm uses alpha, not the current version of alpha1, to update the parental gamete frequencies. Thus, they use the true value of *α* in their code that estimates *α*. This clearly results in unwarranted advantages. Their simulations were rerun after that bug was fixed, obtaining very biased estimates of *α* (Supplementary Appendix S5). This indicates that either their EM algorithm is wrong or their code is incorrect. It is hard to judge if their EM algorithm is wrong since the EM algorithm used to estimate *α* is neither in the article nor in the Supplementary Materials. It is also noted that when the authors’ “estimate_alpha.R” was run, which should produce the simulation results in their Table 3, it was not able to actually reproduce their Table 3.

From what can be understood through their code (in files “table2_power.R” and “LR.R″), Wang et al. (2021) implemented their tests by using the true genotype frequencies when constructing their test statistics. Needless to say, researchers would not have access to the true genotype frequencies in reality. In their “table2_power.R,” they set some genotype frequencies ***q***_1_ and then obtain the underlying true genotype frequencies *via* a perturbation of ***q*** = *f* (***q***_1_, *α*), where *α* = 0. They obtain two equivalently valued variables called prob and prob1. They use a perturbation of prob to generate the data, and prob1 to construct the test statistic, but both prob and prob1 are equal to ***q***. An annotated version of “table2_power.R” is provided in the Supplementary Material so that it is easier for the reader to see the issue here. Though the reader is warned, their code is rather verbose and spans 49 8.5 × 11″ pages. Because their test statistic is impossible to be calculated in real analyses (because it uses the true genotype frequencies), the simulation results of Wang et al. (2021) are invalid. It is also noted that when “table2_power.R” was run using the authors’ original code, it was not able to actually reproduce the power results in their Table 2.

## 3 Distinction between random mating and equilibrium

Sun et al. (2021), Wang et al. (2022), and Wang et al. (2021) suggest that Eqs 1 and 2 are the same hypothesis, or at least approximately so. In their articles, they have a “recursive” test and a “gamete-based” test that they claim both tests for “asymptotic Hardy–Weinberg equilibrium.” Their “recursive” test does indeed evaluate Eq. 2 (assuming *α* is known). However, the “gamete-based” test actually evaluates Eq. 1.

Since the authors say that Eq. 1 is about the same as Eq. 2 for any choice of *α*, this is worth some exploration. As an extreme counterexample (Supplementary Appendix S1), let ***p*** = (0, 0, 1, 0), then hypothesis 1) states that

$$q_{1}=\left(0,0,0,0,1,0,0\right).$$ (5)

But ***q***_1_ is not at equilibrium, and one can use ***q***_1_ as the starting point for many rounds of random mating to reach equilibrium (Eq. 2). When one does, one obtains

$$q_{2}=\left(0.001,0.016,0.082,0.219,0.329,0.263,0.088\right),$$ (6)

when *α* = 0, the lower bound of the double reduction rate. One also obtains

$$q_{3}=\left(0.005,0.032,0.098,0.204,0.277,0.251,0.133\right),$$ (7)

when *α* = 0.3, the upper bound of the double reduction rate (Huang et al., 2019). Clearly, ***q***_1_, ***q***_2_, and ***q***_3_ are very different. But Sun et al. (2021), Wang et al. (2022), and Wang et al. (2021) suggest that they should be about the same.

As a less contrived example, S1 populations (a single generation of selfing) are technically random mating populations, but hardly any researcher would claim that an S1 population is at equilibrium (see Gerard (2022b) for details).

The only real data example used in the study by Wang et al. (2022) consists of four markers from an F1 population. This is insufficient to explore their methods, as F1 populations exhibit neither random mating (Eq. 1) nor equilibrium (Eq. 2). Furthermore, they did not apply their test for random mating on these data, but rather a test for binomial frequencies (Eq. 3), which is a standard approach, though an incorrect one for F1 populations.

## 4 Distinction between allo- and autopolyploids

Wang et al. (2021) stated on page four that “The case of no double reduction in the autopolyploid model reduces to allopolyploids if no preferential pairing is assumed.” Sun et al. (2021) stated on page three that “When *α* = 0, the pattern of allelic inheritance reduces from autotetraploids to allotetraploids.” Since allopolyploids exhibit disomic inheritance within each subgenome (Stift et al., 2010), this is true only if all subgenomes of an allopolyploid have the exact same allele frequency. This is likely not the case in true allopolyploids. In an extreme example, suppose that there is an allooctoploid population with an allele frequency of 0 in two of its subgenomes, and an allele frequency of one in the other two subgenomes; then the overall allele frequency is 0.5, and the allooctoploid equilibrium genotype frequencies are

$$q_{\mathrm{allo}}=\left(0,0,0,0,1,0,0,0,0\right),$$ (8)

because every individual will have two minor alleles each from two subgenomes, and two major alleles each from two subgenomes, and therefore, all individuals will have genotype 4. Compare this to the genotype frequencies of an autooctoploid with allele frequency 0.5 at equilibrium when there is no double reduction

$$q_{\mathrm{auto}}=\left(0.004,0.031,0.109,0.219,0.273,0.219,0.109,0.031,0.004\right).$$ (9)

Clearly, ***q***_*allo*_ and ***q***_*auto*_ are very different. This is not a contrived example, as it might be the case that some subgenomes have fixed an allele before the polyploidization event (“fixed heterozygosity,” Cornille et al., 2016).

The tests created in the studies by Sun et al. (2021), Wang et al. (2022), and Wang et al. (2021) are only applicable to autopolyploids, but the only real data example in the studies by Sun et al. (2021) and Wang et al. (2021) are allopolyploids. So the authors did not adequately evaluate their method on a reasonable dataset.

## 5 Hypothesis testing strategies

The test for equilibrium (Eq. 2) in the studies by Sun et al. (2021), Wang et al. (2021), and Wang et al. (2022) assumes that the double reduction rate is known. But it would not be clear to the reader that this is the case from a reading of the articles. The double reduction rate is never known in practice.

The “recursive” approach in the studies by Sun et al. (2021), Wang et al. (2021), and Wang et al. (2022) for equilibrium testing is unnecessary. The equilibrium frequencies of tetraploids, hexaploids, and octoploids in the presence of double reduction are well documented in the excellent article of Huang et al. (2019). For example, for hexaploids, the equilibrium gamete frequencies are

$$p_{0}=\left(1-\frac{9\left(3-\alpha \right)\left(6-\alpha \right)}{\left(9+\alpha \right)\left(9+2\alpha \right)}r+\frac{27\left(1-\alpha \right)\left(3-\alpha \right)}{\left(9+\alpha \right)\left(9+2\alpha \right)}r^{2}\right)\left(1-r\right),$$ (10)

$$p_{1}=\left(\frac{9\left(3-\alpha \right)\left(9-4\alpha \right)}{\left(9+\alpha \right)\left(9+2\alpha \right)}-\frac{81\left(1-\alpha \right)\left(3-\alpha \right)}{\left(9+\alpha \right)\left(9+2\alpha \right)}r\right)r\left(1-r\right),$$ (11)

$$p_{2}=\left(\frac{45\alpha \left(3-\alpha \right)}{\left(9+\alpha \right)\left(9+2\alpha \right)}+\frac{81\left(1-\alpha \right)\left(3-\alpha \right)}{\left(9+\alpha \right)\left(9+2\alpha \right)}r\right)r\left(1-r\right),\text{ and}$$ (12)

$$p_{3}=\left(\frac{20\alpha^{2}}{\left(9+\alpha \right)\left(9+2\alpha \right)}+\frac{45\alpha \left(3-\alpha \right)}{\left(9+\alpha \right)\left(9+2\alpha \right)}r+\frac{27\left(1-\alpha \right)\left(3-\alpha \right)}{\left(9+\alpha \right)\left(9+2\alpha \right)}r^{2}\right)r.$$ (13)

The equilibrium genotype frequencies are discrete linear convolutions of these proportions. Eqs 10–13 look complicated, but they are not complicated for a computer. It is easy to implement a likelihood approach to test for equilibrium using these gamete frequencies, and such an approach, advantageously, does not depend on knowing the double reduction rate, which is a huge benefit over the iterative approach of Sun et al. (2021), Wang et al. (2021), and Wang et al. (2022). Indeed, this likelihood approach is what was used in the study by Gerard (2022b).

Genotype uncertainty is a major issue in polyploids (Gerard et al., 2018; Gerard and Ferrão, 2019; Gerard, 2021a,b), and so methods should be adjusted to account for this uncertainty. The standard approach to do so is using genotype likelihoods (Li et al., 2011), and this is what was used in the studies by Gerard (2022b) and Gerard (2022a). However, Sun et al. (2021), Wang et al. (2021), and Wang et al. (2022) approach this by aggregating heterozygous genotypes into a single count, which leaves them with only enough degrees of freedom to test for binomial frequencies Eq. 3. They thus provide no way to evaluate hypotheses Eq. 1 and Eq. 2 in the presence of genotype uncertainty.

## 6 Discussion

Here, some implementation mistakes and some misconceptions about the genotype frequencies of autopolyploid organisms presented in the studies by Sun et al. (2021), Wang et al. (2021), and Wang et al. (2022)have been discussed. Examples of how random mating and equilibrium differ in autopolyploids, and also how allo- and autopolyploid equilibrium genotype frequencies differ, have been provided. Finally, it was suggested that users consider the approaches of Gerard (2022b) and Gerard (2022a), which do not assume that the double reduction rate is known, and can account for genotype uncertainty through the use of genotype likelihoods.

Sun et al. (2021), Wang et al. (2021), and Wang et al. (2022) could have averted many of their issues if they would have adhered to standard practices in the validation of numeric analysis and coding. In the future, the authors are encouraged to 1) apply unit testing (Wickham, 2011), 2) set up continuous integration (Hilton et al., 2016), 3) implement code review (Vable et al., 2021), 4) modularize their code into functions, ideally, in a package (Wickham, 2015), 5) use a workflow management software to aid in reproducibility and decrease the chance for coding errors (Blischak et al., 2019), 6) provide instructions (ideally automation) on specifically how to reproduce their methods (Heil et al., 2021), and 7) post their code on a repository that is committed to permanency and produces DOI’s, such as Zenodo or Figshare, as this extends the lifetime of a work’s reproducibility. It is also recommended to encourage greater validation checks, such as demonstrating that the authors’ test statistic produce *p*-values that are uniform under the null. This alone could have detected the test statistic and degrees of freedom issues discussed in Section 2 (Supplementary Appendix S4).

## Data Availability

Additional materials related to this work is available on Zenodo: https://doi.org/10.5281/zenodo.7019205. • The file “hwesupp.Rmd” is an R Markdown file that contains Appendices S1–S6, and is sufficient to reproduce all of the results of this paper. It has been knitted into “hwesupp.pdf”. • The file “sims.csv” contains the simulation output from Appendix S5 of “hwesupp.Rmd”. • The file “table 2_power.Rmd” contains one iteration of “table 2_power.R” from Wang et al., 2021, annotated to demonstrate the mistakes here. It has been knitted into “table 2_power.pdf”. Much of the code from Wang et al., 2021 and Wang et al., 2022 was packaged by me in the hexocto package on Zenodo https://doi.org/10.5281/zenodo.7019230. A fork of the original code from Wang et al., 2021 and Wang et al., 2022 may be found at at https://github.com/dcgerard/hexaploid and https://github.com/dcgerard/OctoploidDeer.
