# Supplementary material for: Comment on three papers about Hardy–Weinberg equilibrium tests in autopolyploids
Source: Front Genet. 2022 Oct 4;13:1027209. doi: 10.3389/fgene.2022.1027209 (PMC9576855; doi:10.3389/fgene.2022.1027209)
Supplement: Supplementary file 1 [file DataSheet1.PDF]

Supplement to: Comment on Three Papers about Hardy-Weinberg Equilibrium Tests in Autopolyploids

David Gerard
Department of Mathematics and Statistics, American University, Washington, DC, 20016, USA

Abstract

This supplementary document contains additional simulations, coding examples, and other supporting material for "Comment on Three Papers about Hardy-Weinberg Equilibrium Tests in Autopolyploids".

This document was written in R Markdown and may be explored interactively. All code chunks are executable in the order given. You can access the R Markdown file at https://doi.org/10.5281/zenodo.7019205.

The package {hexocto} contains the code from Wang et al. (2021) and Wang et al. (2022), formatted in package form by me so that it is easier to compare. The original repos with the original code are https://github.com/CCBBeijing/hexaploid and https://github.com/CCBBeijing/OctoploidDeer. You can install this package using {devtools}:

```
# install.packages("devtools")
devtools::install_github("dcgerard/hexocto")
```

The package {hwep} contains the code from Gerard (2022). I use it for comparison purposes at times. You can install the development version via:

```
# install.packages("devtools")
devtools::install_github("dcgerard/hwep")
```

I will load these packages into R now:

```
library(hexocto)
library(hwep)
```

S1 Difference between random mating and equilibrium

Here, I numerically demonstrate the difference between random mating and equilibrium in autohexaploids.

For illustration, let's make an extreme example. Suppose the gamete frequencies for a hexaploid are

```
p <- c(0, 0, 1, 0)
p
```

```
## [1] 0 0 1 0
```

Then the genotype frequencies under random mating are

```
q <- convolve(p, rev(p), type = "open")
round(q, digits = 3)
```

```
## [1] 0 0 0 0 1 0 0
```

The allele frequency is

```r
r <- sum(0:6 * q) / 6
r
```

```
## [1] 0.667
```

This results in equilibrium frequencies of the following when $\alpha = 0$, the lower bound,

```r
hwep::hwefreq(r = r, alpha = 0, ploidy = 6, niter = Inf)
```

```
## [1] 0.00137 0.01646 0.08230 0.21948 0.32922 0.26337 0.08779
```

I can verify this by iterating the recursive scheme from Wang et al. (2022).

```r
qw <- q
for (i in 1:20) {
  qw <- hexocto::hex_onegen(yww = qw, alpha = 0)
}
qw
```

```
## [1] 0.00137 0.01646 0.08230 0.21948 0.32922 0.26337 0.08779
```

Equilibrium frequencies when $\alpha = 0.3$, the upper bound (Huang et al. 2019), are

```r
hwep::hwefreq(r = r, alpha = 0.3, ploidy = 6, niter = Inf)
```

```
## [1] 0.00537 0.03190 0.09792 0.20350 0.27748 0.25115 0.13268
```

I can also verify this by iterating the recursive scheme from Wang et al. (2022).

```r
qw <- q
for (i in 1:20) {
  qw <- hexocto::hex_onegen(yww = qw, alpha = 0.3)
}
qw
```

```
## [1] 0.00537 0.03190 0.09792 0.20350 0.27748 0.25115 0.13268
```

S2 Incorrect equilibrium genotype frequencies from Wang et al. (2021)

I will begin at the same example genotype frequencies as Wang et al. (2021).

```r
yww <- c(0.1, 0.1, 0.15, 0.1, 0.2, 0.1, 0.05, 0.1, 0.1)
```

Then I apply their recursive approach to obtain their equilibrium genotype frequencies

```r
hexocto::octo_recursive(yww = yww, niter = 20, alpha = 0)
```

```
## [1] 0.0186 0.0677 0.1424 0.2064 0.2234 0.1793 0.1076 0.0443 0.0104
```

These are different from the theoretical binomial proportions Haldane (1930)

```r
r <- sum(0:8 * yww) / 8
dbinom(x = 0:8, size = 8, prob = r)
```

```
## [1] 0.00577 0.04177 0.13228 0.23937 0.27071 0.19594 0.08864 0.02291 0.00259
```

My {hwep} package, on the other hand, correctly calculates these using my recursive formula

```r
qcurrent <- yww
for (i in seq_len(20)) {
  qcurrent <- hwep::freqnext(freq = qcurrent, alpha = c(0, 0))
```

```r
}
qcurrent
```

```
## [1] 0.00577 0.04177 0.13228 0.23937 0.27071 0.19594 0.08864 0.02291 0.00259
```

S3 Coding errors for χ^2 statistics

Wang et al. (2022) use the following as an example for their tests for equilibrium and random mating on page 5 of their manuscript.

```r
nvec <- c(29, 21, 17, 10, 10, 10, 23)
nind <- sum(nvec)
```

Here, I will reproduce those tests, and demonstrate that they implemented their χ^2 test statistics incorrectly.

Their recursive test gets a chi-squared value of 6.602, which I can get here.

```r
hex_chisq(yww = nvec / nind,
          nind = nind,
          niter = 8,
          alpha = 0,
          method = "incorrect")
```

```
## $chisq
## [1] 6.6019
##
## $df
## [1] 6
##
## $p
## [1] 0.35924
```

This is the "incorrect" way because they forgot to account for the number of individuals in the chi-squared test. It should be 120 times 6.602.

```r
# generate their equilibrium frequencies
qhat <- hex_recursive(yww = nvec / nind, niter = 8, alpha = 0)
# does not use nind
sum((qhat - (nvec / nind))^2 / (qhat))
```

```
## [1] 6.6019
```

```r
# correct way
nind * sum((qhat - (nvec / nind))^2 / (qhat))
```

```
## [1] 792.23
```

For the "gamete based test", they get 6.649, but this is not correct. They were just calculating the same test statistic as the 6.602 value, but ran it for a different number of iterations.

```r
# Estimate gamete frequencies
hout <- hex_em(yww = nvec / nind, niter = 30)
# Feed those into recursive algorithm
rvec <- hex_recursive(yww = hout$q, niter = 8, alpha = 0)
# Incorrect way
sum((nvec/nind - rvec)^2 / rvec)
```

```
## [1] 6.6487
```

Here is the value they were trying to get.

```
# Incorrectly does not multiply by nind
sum((nvec/nind - hout$q)^2 / hout$q)
```

```
## [1] 0.30123
```

```
# Correctly multiplies by nind
nind * sum((nvec/nind - hout$q)^2 / hout$q)
```

```
## [1] 36.147
```

The authors' two procedures would produce the same values if you ran them for long enough.

```
# "recursive test" from Wang et al. (2021)
hex_chisq(yww = nvec / nind,
          nind = nind,
          niter = 20,
          alpha = 0,
          method = "incorrect")$chisq
```

```
## [1] 6.7014
```

```
# Implementation of "gamete-based" test from Wang et al. (2021)
hout <- hex_em(yww = nvec / nind, niter = 30)
rvec <- hex_recursive(yww = hout$q, niter = 20, alpha = 0)
sum((nvec/nind - rvec)^2 / rvec)
```

```
## [1] 6.7014
```

This is the exact same as just testing for binomial frequencies, but calculating the χ^2 statistic incorrectly.

```
rhat <- sum(nvec / nind * 0:6) / 6
qhat <- dbinom(x = 0:6, size = 6, prob = rhat)
sum((nvec/nind - qhat)^2 / qhat)
```

```
## [1] 6.7014
```

S4 Correct degrees of freedom

Here, I show that the method Wang et al. (2022) does not produce uniform p-values under the null of equilibrium. I also show that my correct version, including the correct degrees of freedom of 5, not 6, does produce uniform p-values under the null of equilibrium. I also find the correct degrees of freedom for the recursive test in Wang et al. (2021) to be 7, not 8.

S4.1 Hexaploids

I generate data under the null of equilibrium. I then fit the incorrect method Wang et al. (2022), my corrected version, and the likelihood ratio test from Gerard (2022).

```
qvec <- hwep::hwefreq(r = 0.5, alpha = 0.1, ploidy = 6)
nrep <- 1000
nsize <- 100000
pout_wang <- rep(NA_real_, length.out = nrep)
pout_correct <- rep(NA_real_, length.out = nrep)
pout_hwep <- rep(NA_real_, length.out = nrep)
for (i in seq_len(nrep)) {
  nvec <- c(rmultinom(n = 1, size = nsize, prob = qvec))
  pout_wang[[i]] <- hex_chisq(yww = nvec / sum(nvec),
```

```
                              nind = sum(nvec),
                              alpha = 0.1,
                              method = "incorrect")$p
  pout_correct[[i]] <- hex_chisq(yww = nvec / sum(nvec),
                                 nind = sum(nvec),
                                 alpha = 0.1,
                                 method = "correct")$p
  pout_hwep[[i]] <- hwep::hwelike(nvec = nvec, thresh = 0)$p_hwe
}
```

All of the p-values from Wang et al. (2022) are 1, so do not follow a uniform distribution.

```
summary(pout_wang)
```

```
##    Min. 1st Qu.  Median    Mean 3rd Qu.    Max.
##       1       1       1       1       1       1
```

The QQ-plot of the correct p-values follow a uniform distribution.

```
library(ggplot2)
qplot(sample = pout_correct, geom = "qq", distribution = qunif) +
  geom_abline()
```

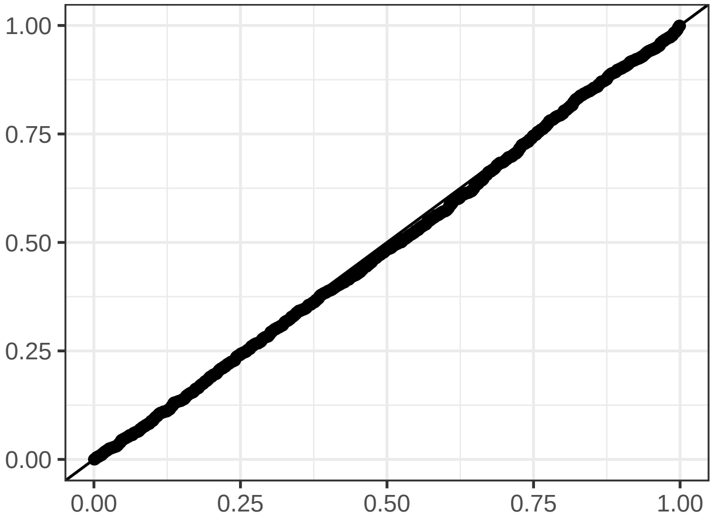

The QQ-plot of the {hwep} p-values follow a uniform distribution.

```
qplot(sample = pout_hwep, geom = "qq", distribution = qunif) +
  geom_abline()
```

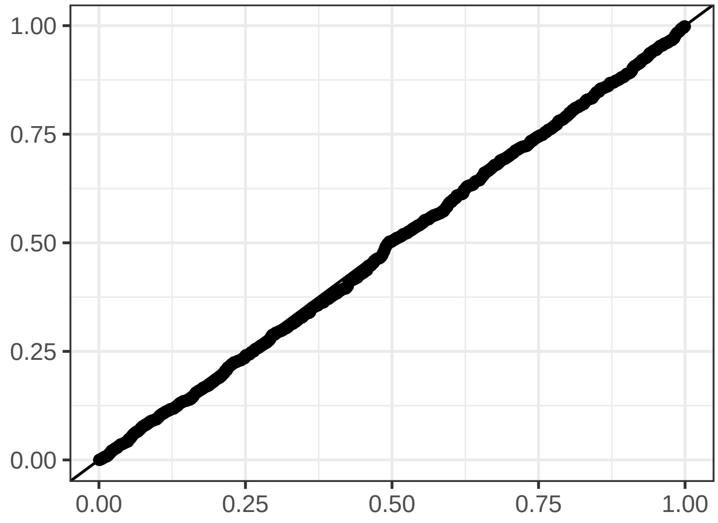

S4.2 Octoploids

I generate data under the incorrect model of Wang et al. (2021), calculating the χ^2 statistic each iteration.

```
yww1 <- c(0, 0, 0, 0, 1, 0, 0, 0, 0)
qvec <- octo_recursive(yww = yww1)
nrep <- 1000
nsize <- 100000
chstat_octo <- rep(NA_real_, length.out = nrep)
for (i in seq_len(nrep)) {
  nvec <- c(rmultinom(n = 1, size = nsize, prob = qvec))
  qemp <- nvec / sum(nvec)
  qnew <- octo_recursive(yww = qemp)
  chstat_octo[[i]] <- sum((qnew - qemp)^2 / qnew) * sum(nvec)
}
```

The degrees of freedom is not 8:

```
pocto <- pchisq(q = chstat_octo, df = 8, lower.tail = FALSE)
qplot(sample = pocto, geom = "qq", distribution = qunif) +
  geom_abline()
```

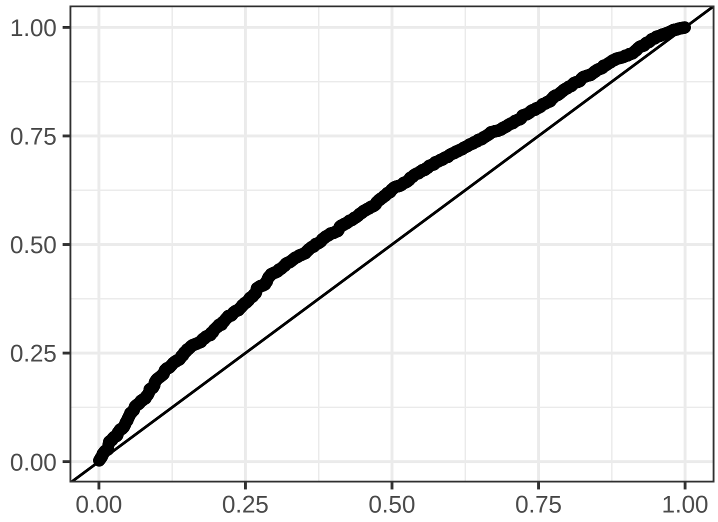

The degrees of freedom *is* 7:

```
pocto <- pchisq(q = chstat_octo, df = 7, lower.tail = FALSE)
qplot(sample = pocto, geom = "qq", distribution = qunif) +
  geom_abline()
```

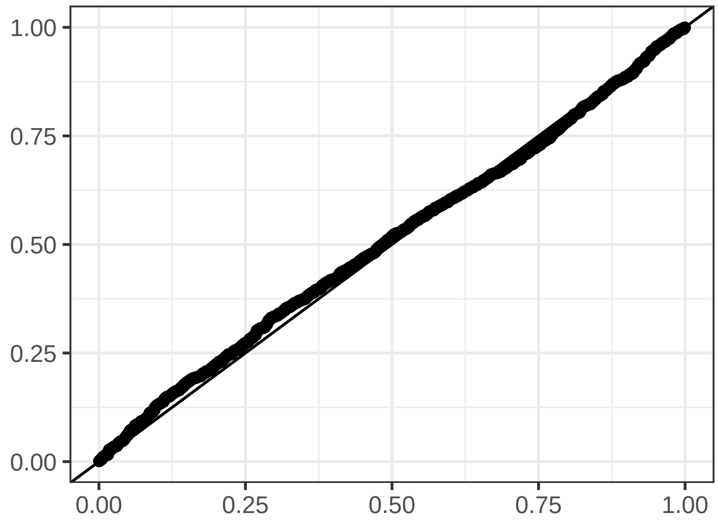

S5 Simulation study to estimate α

The model Wang et al. (2022) used to create an estimator for α is actually different from (1) and (2). Their model to estimate double reduction says that (i) parent genotypes frequencies satisfy $\tilde{\boldsymbol{q}} = \boldsymbol{p} * \boldsymbol{p}$ for some $\boldsymbol{p}$, and (ii) the current genotype frequencies are $\boldsymbol{q} = f(\tilde{\boldsymbol{q}}, \alpha)$. So this indicates random mating for parents, and one update of random mating for children given the double reduction rate.

I ran simulations with $\boldsymbol{p} = (1, 1, 1, 1)/4$ or $\boldsymbol{p} = (0.1, 0.2, 0.3, 0.4)$, $n \in \{100, 200, 400\}$, and $\alpha \in \{0, 1/7, 1/5, 3/11\}$. This mimics the simulation settings from Wang et al. (2022). I ran each unique combination of parameter settings for 100 replications. Each replication, I generated data according to the assumed model from Wang et al. (2022), then used their code to obtain estimates of $\boldsymbol{p}$ and α. I always initialized the algorithm at $\alpha = 0$ and $\boldsymbol{p} = (1, 1, 1, 1)/4$.

Below is my simulation code.

```
## Parameter settings ----
pvec1 <- rep(1, 4) / 4
qvec1 <- convolve(pvec1, rev(pvec1), type = "open")
pvec2 <- c(0.1, 0.2, 0.3, 0.4)
qvec2 <- convolve(pvec2, rev(pvec2), type = "open")

niter <- 100
paramdf <- expand.grid(seed = seq_len(niter),
                       n = c(100, 200, 400),
                       alpha = c(0, 1/7, 1/5, 3/11),
                       truth = c("A", "B"))

## Estimates to fill in ----
paramdf$alphahat <- NA_real_
paramdf$p0hat <- NA_real_
paramdf$p1hat <- NA_real_
paramdf$p2hat <- NA_real_
paramdf$p3hat <- NA_real_
```

```r
## Simulations ----
for (i in seq_len(nrow(paramdf))) {
  set.seed(paramdf$seed[[i]])
  ## offspring genotype frequencies
  if (paramdf$truth[[i]] == "A") {
    qoff <- hex_onegen(yww = qvec1, alpha = paramdf$alpha[[i]])
  } else {
    qoff <- hex_onegen(yww = qvec2, alpha = paramdf$alpha[[i]])
  }

  ## sample of offspring
  nvec <- c(rmultinom(n = 1, size = paramdf$n[[i]], prob = qoff))

  ## estimate parameters
  hout <- hex_estdr(NN = nvec, niter = 1000, tol = 0)
  paramdf$alphahat[[i]] <- hout$alpha
  paramdf$p0hat[[i]] <- hout$p[[1]]
  paramdf$p1hat[[i]] <- hout$p[[2]]
  paramdf$p2hat[[i]] <- hout$p[[3]]
  paramdf$p3hat[[i]] <- hout$p[[4]]
}
write.csv(x = paramdf, file = "./sims.csv", row.names = FALSE)
```

The estimates of α are very biased (Figure S1), and the estimates of $\boldsymbol{p}$ are somewhat biased (Figure S2).

S6 Degrees of Freedom Calculations

Here, I list out the five instances of incorrect degrees of freedom calculations from Sun et al. (2021), Wang et al. (2022), and Wang et al. (2021).

The degrees of freedom for the both the equilibrium and random mating tests are incorrect in Sun et al. (2021). They list the degrees of freedom to be four in both tests. But there are already four free parameters under the alternative (since $q_0 + q_1 + q_2 + q_3 + q_4 = 1$). Since Sun et al. (2021) assume the double reduction rate is known, under the null of equilibrium there is one free parameter (the allele frequency), and so the degrees of freedom for the test for equilibrium is $4 - 1 = 3$, not 4. Under the null of random mating, there are 2 free parameters (since $p_0 + p_1 + p_2 = 1$), and so the degrees of freedom for the test of random mating is $4 - 2 = 2$, not 4.

The degrees of freedom for the random mating test is incorrect in Wang et al. (2022). On page 4 of Wang et al. (2022), the authors say about their test for random mating that "this test statistic follows the chi-square distribution with an unknown degree of freedom. However, we can empirically determine it as a value between 7 - 1 - 1 = 5 to 7 - 1 = 6." I can theoretically determine the degrees of freedom here. There are 6 free parameters under the alternative (since $q_0 + q_1 + q_2 + q_3 + q_4 + q_5 + q_6 = 1$), and there are 3 free parameters under the null (since $p_0 + p_1 + p_2 + p_3 = 1$), and so the degrees of freedom is 6 - 3 = 3, which is neither 5 nor 6.

The degrees of freedom for the recursive test is incorrect in Wang et al. (2022). They say, right after their equation (1) that the degrees of freedom is 6. But there are already 6 free parameters under the alternative. Because Wang et al. (2022) assume the double reduction rate is known, there is only 1 free parameter under the null, the allele frequency. Thus, the true degrees of freedom is 6 - 1 = 5, not 6. See Appendix S4 for an empirical demonstration.

The degrees of freedom for the recursive test is incorrect in Wang et al. (2021). Right after their equation (3), they state that their χ^2 statistic "is thought to follow a chi-square distribution with eight degrees of freedom." But there are already 8 parameters under the alternative (since $\sum_{k=0}^{8} q_k = 1$). The number of parameters

under the null is unclear since they are using a different (incorrect) model for meiosis than I have studied for octoploids, but it likely at least 1 (for the allele frequency). Empirically, it seems the degrees of freedom is 7, not 8 (Appendix S4).

S7 Supplementary Figures

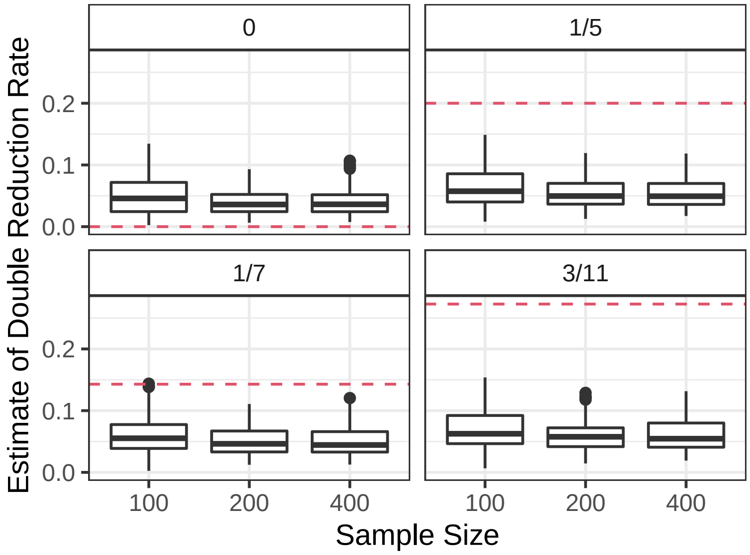

Figure S1: Estimates of α (y-axis) stratified by sample size (x-axis) and true α (facets) using the method of Wang et al. (2022). The red dashed horizontal line is the true α in each facet. The estimates are way off.

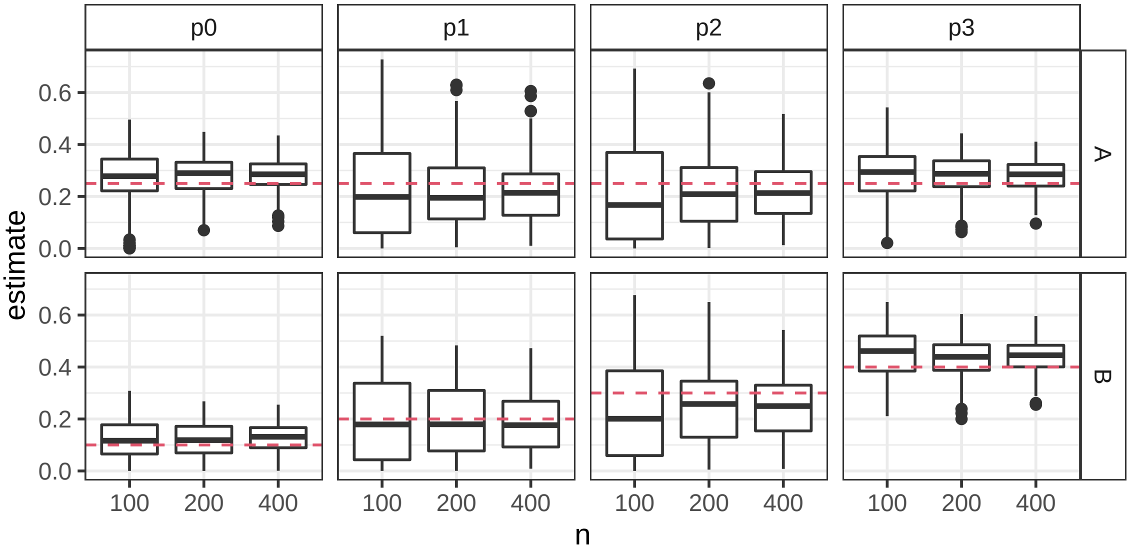

Figure S2: Estimates of p_k (y-axis) for $k = 0, 1, 2, 3$ (row facets) for different sample sizes (x-axis) and different initial values (truth or random) using the method of Wang et al. (2022). The red dashed horizontal line is the true p_k in each facet. The estimates are somewhat biased.

References

Gerard, David. 2022. "Double Reduction Estimation and Equilibrium Tests in Natural Autopolyploid Populations." *Biometrics*. https://doi.org/10.1111/biom.13722.

Haldane, JBS. 1930. "Theoretical Genetics of Autopolyploids." *Journal of Genetics* 22 (3): 359–72. https://doi.org/10.1007/BF02984197.

Huang, Kang, Tongcheng Wang, Derek W Dunn, Pei Zhang, Xiaoxiao Cao, Rucong Liu, and Baoguo Li. 2019. "Genotypic Frequencies at Equilibrium for Polysomic Inheritance Under Double-Reduction." *G3: Genes |Genomes |Genetics* 9 (5): 1693–1706. https://doi.org/10.1534/g3.119.400132.

Sun, Lidan, Jingwen Gan, Libo Jiang, and Rongling Wu. 2021. "Recursive Test of Hardy-Weinberg Equilibrium in Tetraploids." *Trends in Genetics* 37 (6): 504–13. https://doi.org/10.1016/j.tig.2020.11.006.

Wang, Jing, Li Feng, Shuaicheng Mu, Ang Dong, Jinwen Gan, Zhenying Wen, Juan Meng, Mingyu Li, Rongling Wu, and Lidan Sun. 2022. "Asymptotic tests for Hardy-Weinberg equilibrium in hexaploids." *Horticulture Research* 9. https://doi.org/10.1093/hr/uhac104.

Wang, Jing, Xuemin Lv, Li Feng, Ang Dong, Dan Liang, and Rongling Wu. 2021. "A Tracing Model for the Evolutionary Equilibrium of Octoploids." *Frontiers in Genetics* 12. https://doi.org/10.3389/fgene.2021.794907.
